# Hypoxia-induced circRNAs encoded by *PPARA* are highly expressed in human cardiomyocytes and are potential clinical biomarkers of acute myocardial infarction

**DOI:** 10.1186/s40001-024-01753-3

**Published:** 2024-03-12

**Authors:** Shasha Huang, Zhangying Wu, Yang Zhou

**Affiliations:** 1 Department of Cardiology, Shenzhen People’s Hospital (The Second Clinical Medical College, Jinan University, The First Affiliated Hospital, Southern University of Science and Technology), Shenzhen, 518020 , Guangdong China; 2 Department of Cardiology, Qingdao Huangdao District People’s Hospital, Qingdao, 266400 Shangdong China; 3 Department of Central Laboratory, Zhongshan Hospital of Xiamen University, School of Medicine, Xiamen University, No. 201-209, Hubinnan Road, Siming District, Xiamen, 361004 Fujian China

**Keywords:** Acute myocardial infarction, *PPARA*, circRNAs, Peripheral blood, Biomarker

## Abstract

**Background:**

Acute myocardial infarction (AMI) is a serious cardiovascular disease that adversely affects human health. Circular RNAs (circRNAs) are involved in the pathological and physiological processes of AMI, but the biological mechanism of their involvement and their clinical significance remain unknown. We aimed to identify circRNAs that are significantly associated with morbidity in the peripheral blood of patients with AMI and evaluate their diagnostic utility.

**Methods:**

High-throughput sequencing was used to screen for differentially expressed circRNAs in peripheral blood samples obtained from five patients with AMI and five sex- and age-matched healthy controls. A series of bioinformatics tools and databases were used to determine the biological functional classification and pathway enrichment of the circRNAs based on data obtained from sequencing. A hypoxia model was established and used to evaluate the effect of hypoxia on circRNA expression in human cardiomyocytes. A cytoplasmic separation assay and enzyme resistance assay were employed to identify the biological characteristics of circRNA. Polymerase chain reaction validity testing and receiver operating characteristic (ROC) curve analysis were used to evaluate the utility of circRNA assessments in the diagnosis of AMI.

**Results:**

A large number of circRNAs were found to be differentially expressed in the peripheral blood of patients with AMI, and significantly more of these circRNAs were highly expressed than lowly expressed. The genes encoding these circRNAs have a wide range of effects on various functions in the body. A hypoxic environment promoted the upregulation of circRNA expression in human cardiomyocytes, and hsa_circ_0116795 encoded by *PPARA* was highly expressed in the peripheral blood of the patients with AMI. In terms of biological characteristics, under physiological conditions, hsa_circ_0116795 (circ_PPARA) was mainly located in the cytoplasm of cardiomyocytes and found to be resistant to exonuclease. The ROC curve analysis showed that the expression levels of circ_PPARA in the peripheral blood of patients with AMI were significantly different from those in the peripheral blood of healthy controls.

**Conclusion:**

A large number of abnormally expressed circRNAs are detectable in the peripheral blood of patients with AMI. In particular, circ_PPARA is highly expressed in human myocardial cells under hypoxic conditions, and its biological characteristics indicate that it could be employed as a biomarker for the early diagnosis of AMI.

**Supplementary Information:**

The online version contains supplementary material available at 10.1186/s40001-024-01753-3.

## Introduction

Acute myocardial infarction (AMI) is a disease in which myocardial cells are unable to obtain the oxygen they require for metabolism from the coronary artery because coronary blood flow has decreased or ceased, which results in the destruction of myocardial cells [1]. Moreover, if patients with AMI do not receive timely treatment, they may experience heart failure or sudden death [2]. In China, approximately 3.5 million people die from cardiovascular diseases every year, including approximately 2.5 million patients with AMI [3]. Epidemiological data from recent years show that the incidence rate of myocardial infarction in China is gradually increasing and thus represents a serious threat to the health of the population [4]. Furthermore, data from recent years also show that the incidence of AMI is increasing in young people [5]. This is mostly seen in patients with hypertension, diabetes and other diseases or those who have an unhealthy diet, are smokers or have other long-term bad habits [2]. First aid must be provided as soon as possible for patients after they have had a myocardial infarction, but approximately a quarter of such patients do not exhibit any clinical symptoms [1, 4]. In addition, although AMI-related biomarkers such as creatine kinase isoenzyme, serum myoglobin, and cardiac troponins I and T have been applied in clinical practice, their utility is significantly limited [6–8]. For example, although increases in myoglobin concentrations occur relatively early during an AMI, the specificity of this change is not high. This is because myoglobin is present in skeletal muscle cells in addition to myocardial cells, and myoglobin concentrations sometimes increase when skeletal muscle cells are damaged [6]. Therefore, it is particularly important to search for novel biomarkers of AMI.

Circular RNAs (circRNAs) are among the many transcriptional components of genes. They lack the characteristic poly(A) tail molecule of linear transcripts and thus form cyclic structures by connecting end to end [9–11]. Recent studies have shown that the biological properties and functions of circRNAs are different from those of linear RNAs. For example, circRNAs can bind to various microRNAs (miRNAs), thereby isolating them to prevent them from interacting with a target mRNA, i.e. acting as miRNA sponges [11, 12]. Several miRNAs have been found to be associated with AMI and participate in multiple pathophysiological processes of its pathogenesis [6, 13–22]. CircRNAs can also co-function with miRNAs through a competing endogenous RNA mechanism. The closed ring structure of circRNAs means that they are not easily degraded by RNA exonuclease, and they have high expression abundance and a certain extent of timing and disease specificity [9, 12]. As such, circRNAs have great potential to become a new biomarker for the diagnosis of AMI.

The development of new sequencing technologies has led to the discovery of novel circRNAs, some of which have been shown to play an important role in the development and progression of AMI [12, 23]. However, research on circRNAs and their importance remains in its infancy, and many functional explorations have yet to be carried out. In this study, we identified multiple circRNAs that were abnormally expressed in patients with AMI by high-throughput sequencing of total RNA from the peripheral blood of such patients and healthy individuals. Subsequently, to enhance our understanding of the pathogenesis and pathological progression of AMI, we explored the biological characteristics of these circRNAs and their potential utility in the diagnosis of AMI.

## Methods

### Participants

Fifty patients with AMI were recruited from the Central Laboratory of Zhongshan Hospital, Xiamen University, China. A diagnosis of AMI was confirmed by angiography and electrocardiogram. In addition, these patients were subjected to emergency myocardial enzyme profiling to identify evidence of myocardial cell damage. The diagnosis of each patient was independently confirmed by two independent cardiologists who had no prior knowledge of the patients’ medical histories. The diagnostic criteria for AMI followed the guidelines of the American Heart Association. Fifty healthy controls who were sex- and age-matched with the patients with AMI were recruited from the physical examination centre of Zhongshan Hospital. The exclusion criteria were a history of heart disease or hepatitis, liver failure, renal impairment, bleeding disorders, or tumours. The study was performed in accordance with the Declaration of Helsinki and was approved by the Medical Ethics Committee of Zhongshan Hospital. Informed consent was obtained from all of the participants.

### Clinical specimen collection and RNA isolation

Standard venipuncture techniques were used to collect blood into dipotassium ethylenediaminetetraacetic acid anticoagulant vacuum-collection vessels within 2 h of a patient’s diagnosis of AMI. Next, each blood collection vessel was connected to a blood RNA storage tube, and then another blood collection needle was inserted into the cover of the blood collection vessel. Consequently, due to the negative pressure in the storage tube, 2.5 mL of blood flowed into the blood RNA storage tube. The blood RNA storage tube was then inverted and de-inverted vigorously 15–30 times or shaken for more than 60 s to ensure that the blood was fully mixed with the storage solution. The resulting fully mixed sample was allowed to stand at room temperature for 10 min and then subjected to the extraction procedure detailed as follows or stored at an appropriate temperature. For the extraction, a sample was first treated with chloroform, and the resulting mixture was vortexed and then centrifuged to separate the water (top) layer, which contained RNA, from the organic (bottom) layer. Next, the top layer was collected and then treated with isopropyl alcohol to precipitate the RNA.

### High-throughput sequencing of circRNAs in peripheral blood

Total RNA was extracted from the peripheral blood samples of five patients diagnosed with AMI and five healthy controls using the above-described method and then analysed for quality control using an Agilent Bioanalyzer 2100. Next, the total RNA of the five patients with AMI was combined and that of the healthy controls was also combined to create a single RNA sample for each of the two groups of participants. The standard Illumina process was used to construct the circRNA library for de-linearisation of RNA samples, and high-throughput sequencing was conducted on the BGI HiSeq4000 platform to afford a data volume of equal to or greater than 10G.

Ribonuclease R (RNase R) is an exonuclease that digests RNA by progressively cutting it in the 3′ to 5′ direction. It can easily digest most linear RNAs but cannot easily digest circRNAs, lasso structures, or some double-stranded RNAs. Therefore, RNase R is often used to digest linear RNAs in samples and thereby enrich them in circRNAs or lariat RNAs, which is often required for preparing high-throughput sequencing samples. In this study, the total RNA samples were treated successively with RNase R and the Human Ribo-Zero™ Magnetic Gold Kit according to the manufacturer’s instructions. Then, the ribosomal RNA was removed from total RNA.

### Bioinformatics analysis

The total RNA obtained from the above-described procedure was used to construct a complementary DNA (cDNA) library. First, reads of low quality, with contaminated joints, and a high content of unknown base N were filtered out using SOAPnuke to afford clean reads. Next, circRNA prediction was performed using CircRNA Identifier (CIRI) and find_circ, and then differential circRNA were detected using the DEGseq algorithm. Subsequently, the clean reads were compared with the reference genome, and CIRI and find_circ were used to predict circRNAs. The results obtained from the above-described analyses were merged, and then circRNAs were subjected to quantitative and differential expression analysis, and the genes from which circRNAs originated were annotated using the Non-Redundant Protein Sequence, Gene Ontology (GO), and Kyoto Encyclopedia of Genes and Genomes (KEGG) databases [24]. Finally, GO functional enrichment analysis and pathway functional enrichment analysis were performed for genes generated differentially expressed circRNAs. The subcellular localisation of circRNAs was determined using the online analysis tool lncLocator (http://www.csbio.sjtu.edu.cn/bioinf/lncLocator/).

### Detection of circRNAs and linear gene expression

The expression levels of circRNAs and linear mRNAs were detected using the isolated total RNA as the template, and ReverTra Ace^®^ qPCR RT Master Mix (where qPCR = quantitative polymerase chain reaction, and RT = reverse transcription) as the cDNA template transcription system with a total system volume of 10 μL. This reverse transcription system contained 2 μL of 5 × ReverTra Ace qPCR RT Master Mix, 1 μg of total RNA, and an appropriate volume of RNase-free water. Next, the system was mixed by gentle microcentrifugation and then subjected to reverse transcription by PCR at 37 ℃ for 15 min, 50 ℃ for 5 min, and finally 98 ℃ for 5 min. Subsequently, 150 μL of cDNA product was diluted with enzyme-free water and then used directly for the detection of relative quantitative gene expression or frozen at – 20 ℃. Detection was performed by combining, on ice and in triplicate, 8 µL of the diluted cDNA product with 10 µL of PowerUp™ SYBR™ Green Master Mix reagent, 1 µL of specific forward primers (10 µM), and 1 µL of reverse primers (10 µM) of the target gene or circRNAs. The resulting reaction system was gently mixed by microcentrifugation and then placed in an RT-qPCR instrument (CFX96™ Real-Time System,Bio-Rad, CA) for detection. All primer sequence information can be found in Additional file 1: Table S1.

### Cell culture

Human cardiomyocyte cells (AC16; Procell, Wuhan, China) were cultured in an incubator in Dulbecco’s modified Eagle medium (DMEM)–Nutrient Mixture F12 (including 4-(2-hydroxyethyl)-1-piperazineethanesulfonic acid) medium containing 10% foetal bovine serum and 1% penicillin–streptomycin solution at 37 °C and under an atmosphere of 5% carbon dioxide (CO_2_). In the cellular hypoxia model, AC16 cells were cultured in DMEM without foetal bovine serum and with glucose at 37 °C under an atmosphere of 1% oxygen, 5% CO_2_, and 94% nitrogen for 24 h.

### Biological characterisation of circRNAs

The total RNA was digested at 37 °C for 10 min with RNAse R (with 3 U RNase R per microgram of RNA) and then used as a template to synthesise cDNA. Subsequently, the expression levels of linear *PPARA* and circ_PPARA were compared with the expression of *PPARA* and circ_PPARA from conventional cDNA. As cellular sublocalisation often determines the biological function performed by a given circRNA, we next conducted cellular sublocalisation analysis of the selected circRNA using a Cytoplasmic and Nuclear RNA Purification Kit (Norgen Biotek, Canada), according to the standard procedures in the instruction manual.

### PCR validity analysis

The total RNA of AC16 cells was diluted to 100, 50, 25, 12.5 and 6.25 μg/mL, respectively. The total RNA was reverse transcribed to generate cDNA, and then circ_PPARA was amplified using cDNA as template. The linear relationship between the threshold cycle (Ct) value and the logarithm of RNA concentration was also analysed.

### Receiver operating characteristic (ROC) curve analysis

An ROC curve is a common logistic regression model used to assess the sensitivity and specificity of clinical indicators. The value of the area under an ROC curve (AUC) is used to indicate the performance of a classifier and is usually between 0.5 and 1.0. Thus, the expression of *PPARA*-derived circRNA was divided into a healthy control group and a patients with AMI group, and the clinical diagnosis of a patient was set as the true criterion in the model.

### Statistical analysis

All of the experimental results were obtained in triplicate and continuous variables are expressed as means ± standard deviations. A *t*-test was used to compare the data between the two groups, and a one-way analysis of variance combined with multiple comparisons was used to analyse the data of three or more groups. A non-parametric test was used to analyse the data that did not conform to a normal distribution. All statistical plots were generated using GraphPad Prism 9.0 (GraphPad Software, San Diego, CA, USA), and statistical analyses were performed using Statistical Product and Service Solutions 19.0 (IBM Corporation, Armonk, NY, USA). A *P* value of less than 0.05 was considered to indicate a statistically significant difference.

## Results

### Filtering of sequencing data of circRNAs in peripheral blood

**Fig. 1:**
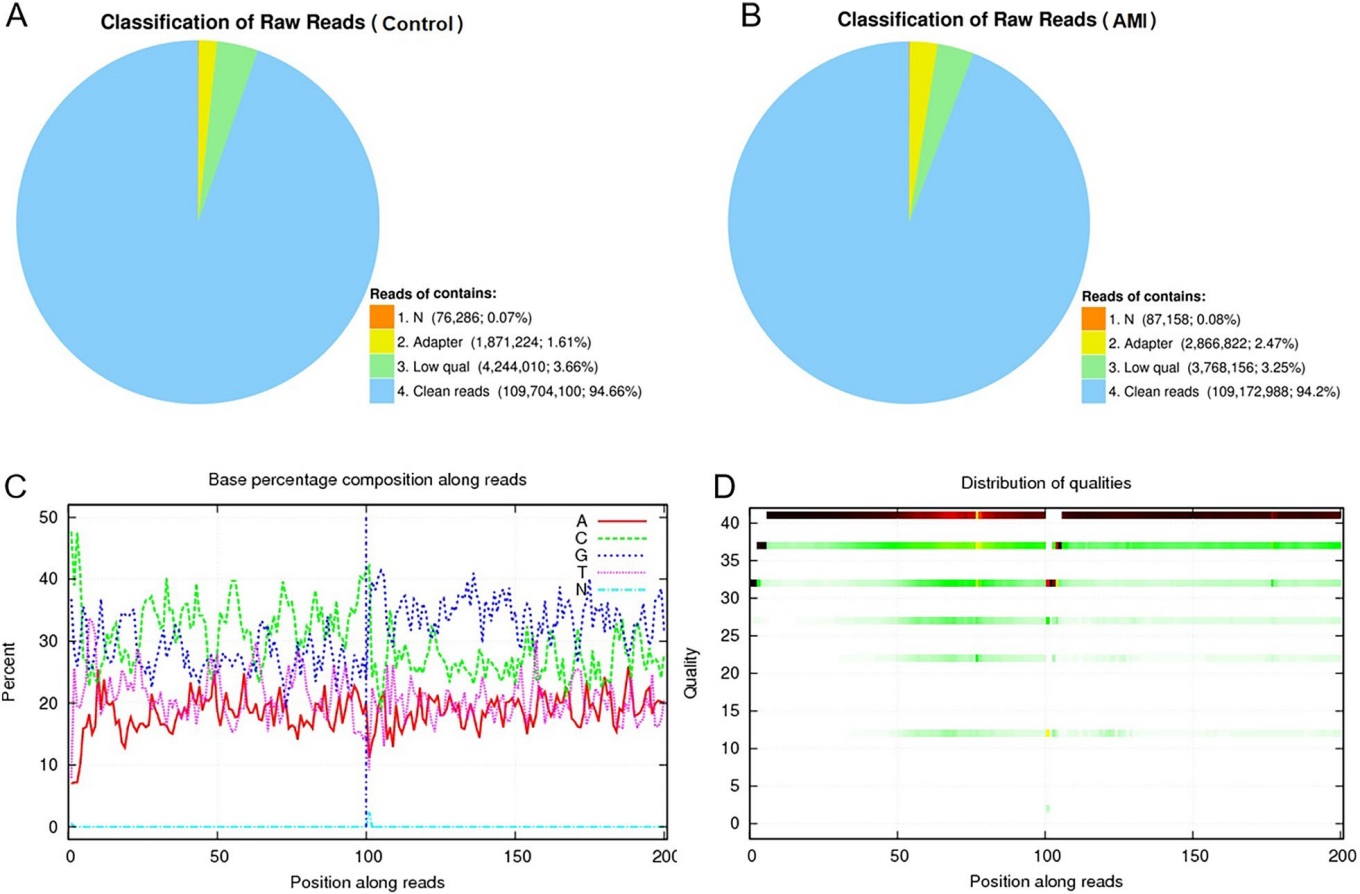
Filtering of high-throughput sequencing data of circRNAs in peripheral blood. Raw data from sequencing included reads that were of low quality, had contaminated joints, and/or had high levels of unknown base N. The statistics of filtering components of each of the two groups of original data are shown. N: the number of reads with an unknown base content greater than 5% and their proportion with respect to the total number of raw reads. Adapter: the number of reads containing the connector (contaminated reads from the connector) and their proportion with respect to the total number of raw reads. Low qual: the number and proportion of low-quality reads (reads with a mass value of less than 15 that account for more than 20% of the total base of the reads). Clean reads: the number of filtered reads and their proportion with respect to the total number of raw reads. **A** Statistical chart of components filtered from the raw data of the healthy control group. **B** Statistical chart of components filtered from the raw data of the AMI group. **C** Distribution map of base content in clean reads. **D** Base mass distribution of clean reads

The original sequencing data of circRNAs in the peripheral blood of the healthy controls and the patients with AMI included reads of low quality with contaminated junctions and with a high content of unknown base N. The statistics of components filtered from the original data are shown in Fig. 1A, B. These non-clean reads were removed before data analysis to ensure the reliability of the results. The base content distribution and quality distribution are shown in Fig. 1C, D.

### Prediction and annotation of circRNA

**Fig. 2:**
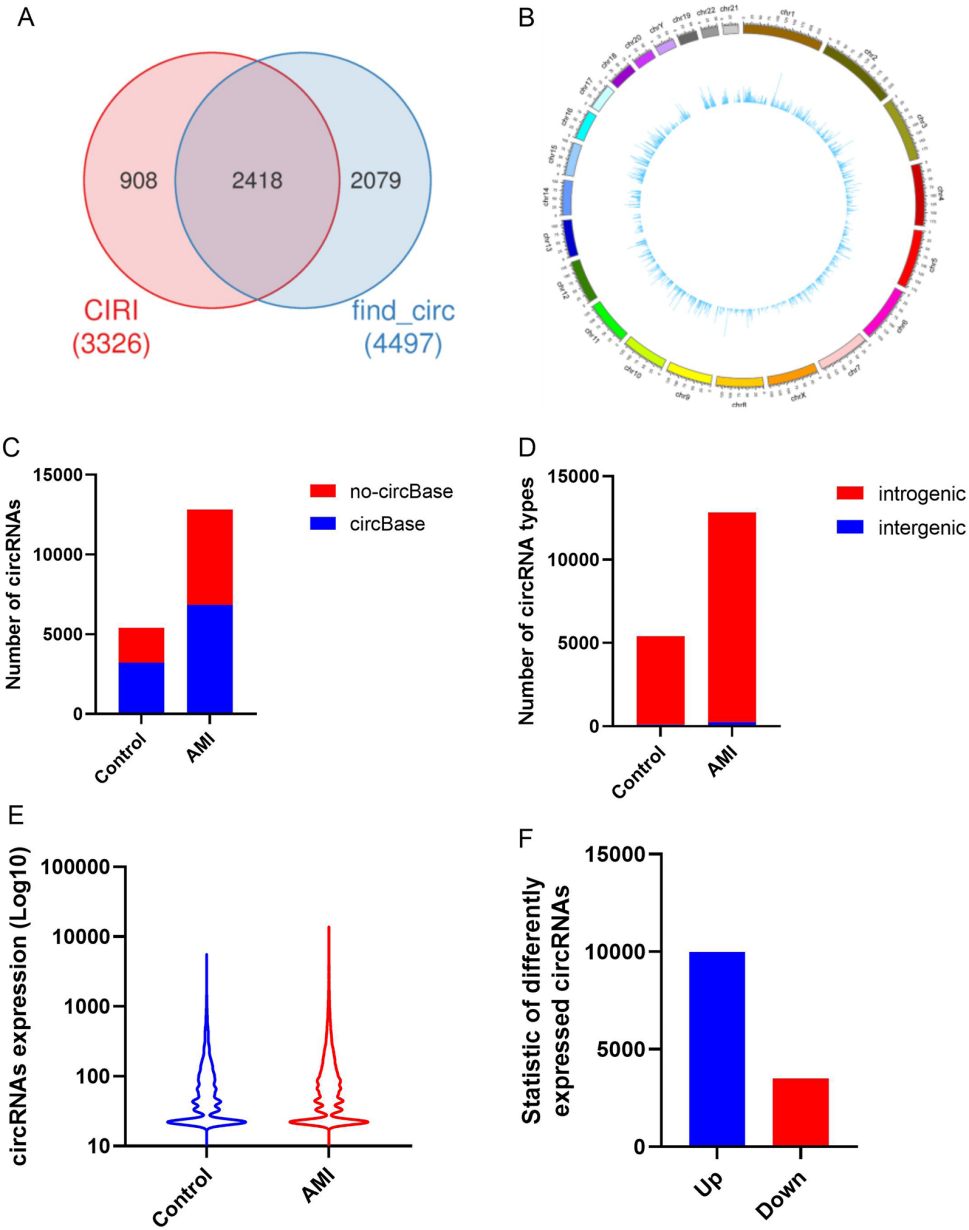
Prediction of circRNA and annotation of biological information. **A** Venn statistical graph of circRNA predicted by CIRI and find_circ. **B** Distribution map of the circRNAs on the genome. **C** Statistics of the number of circRNAs annotated and unannotated by circBase. **D** Column chart of statistics of of types of circRNA. **E** Analysis of the expression level of circRNAs. **F** Statistical chart of the number of different circRNAs

CIRI and find_circ were used to predict circRNA, and the results generated by these two software were integrated (Fig. 2A) according to the starting and ending positions of circRNA (i.e. circRNAs whose starting and ending positions were within the first and last 10 bases were combined into one class). In addition, Circos software was used to show the distribution of circRNAs on the genome, as shown in Fig. 2B. The specific number of circRNAs included by circBase is shown in Fig. 2C. The circRNAs were classified as intragenic circRNAs or intergenic circRNAs according to their starting and ending positions on chromosomes (Fig. 2C). The types of circRNAs in each sample are shown in Fig. 2C. GO, KEGG, NR and other database annotations were determined for the source genes of intragenic circRNAs. As shown in Fig. 2E, the expression of circRNAs in patients with AMI was significantly higher than that in the healthy controls. In addition, in Fig. 2F, the statistics of the number of differentially expressed circRNAs reveal that the expression level of most circRNAs was upregulated.

### The expression of circRNA was significantly different in the peripheral blood of patients with AMI compared with that in the peripheral blood of healthy controls

**Fig. 3:**
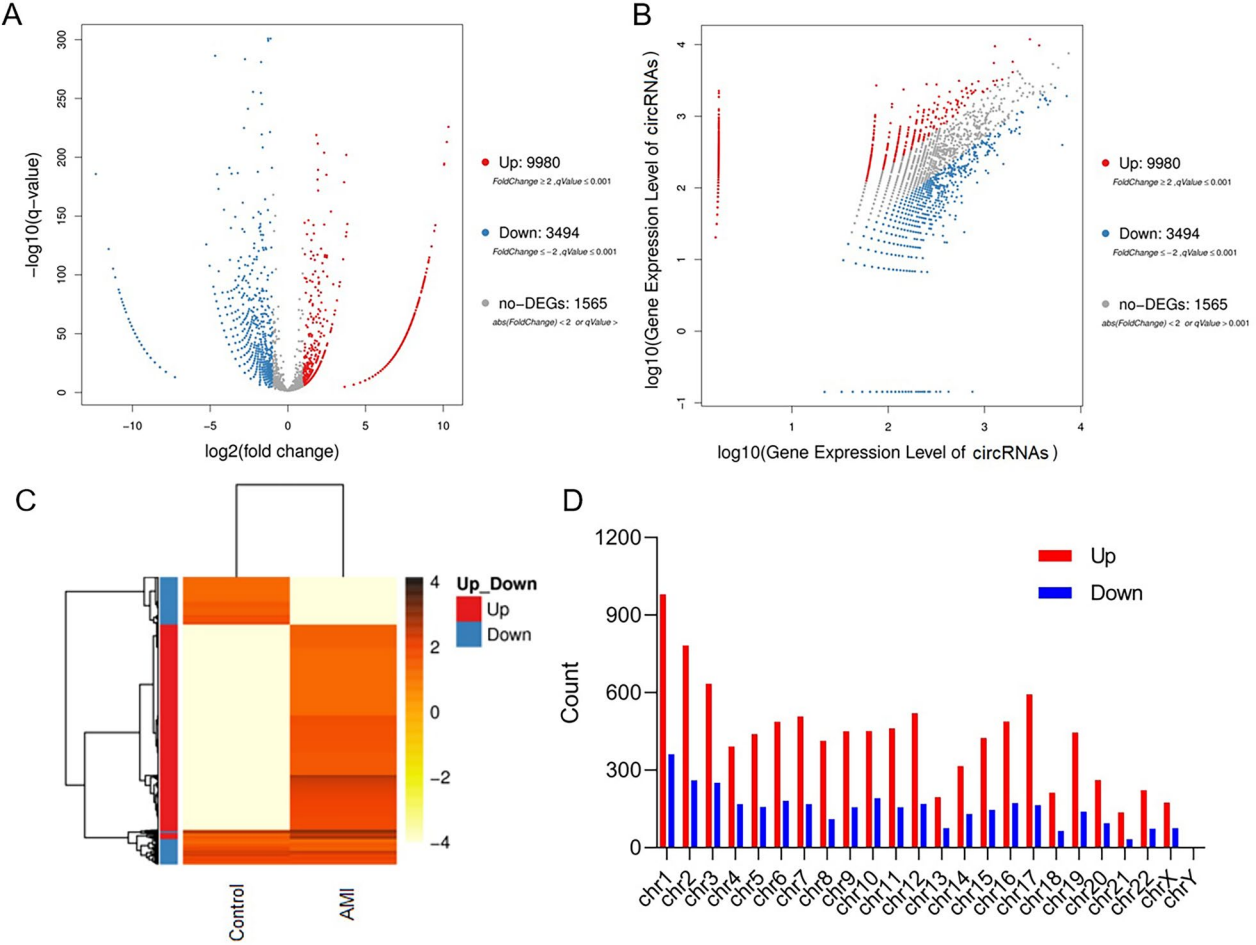
Detection of differential expression of circRNA between healthy controls and patients with AMI. **A** Volcano plot distribution map of differentially expressed circRNAs. **B** Scatter plot distribution of differentially expressed circRNAs. **C** Heat map of differential expression levels of circRNAs. **D** Distribution of differentially expressed circRNAs in human chromosomes

The difference in circRNA expression between the patients with AMI and healthy controls was calculated according to the expression level of circRNA in each sample. The DEGseq method was used to detect differential expression, and a volcano map, scatter map, and heat map were used to display the statistical results (Fig. 3). The identity of all differential circRNAs obtained from sequencing is shown in Additional file 1: Table S2. The results indicate that in the patients with AMI, 9980 circRNAs were highly expressed, 3494 circRNAs were lowly expressed, and 1565 circRNAs were not significantly differentially expressed compared with the healthy controls (Fig. 3). Furthermore, we analysed the distribution of the cDNA of differentially expressed circRNAs on human chromosomes and found that they were present on all but the Y chromosome (Fig. 3D).

### GO functional analysis of genes generated differentially expressed circRNAs

**Fig. 4:**
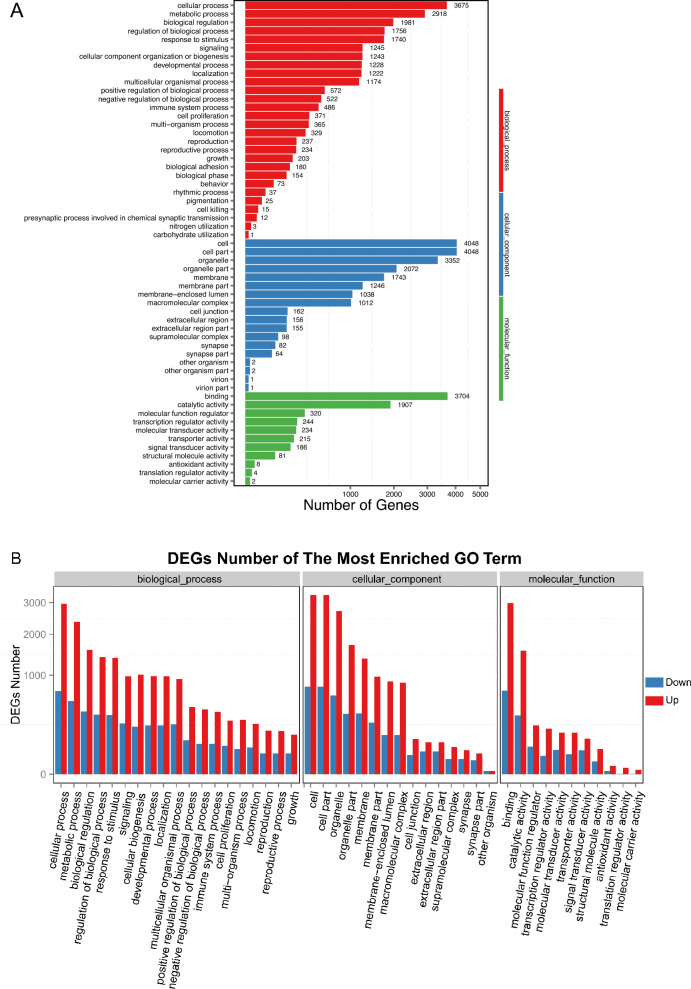
GO functional analysis of genes that differentially expressed circRNAs.** A** GO functional classification of genes that differentially expressed circRNA.** B** GO functional classification statistics of genes that drove the differential expression of circRNAs

Based on the results of the differential expression analysis, a GO functional classification and an enrichment analysis were carried out on the genes generated intragenic circRNAs. GO divides genes into three functional categories: molecular function, cellular component, and biological process. In this study, the three functional categories were further classified and analysed. The GO functional classification results are shown in Fig. 4A, and the GO functional classification statistics of genes generated up- or downregulated circRNAs are shown in Fig. 4B.

### Pathway function analysis of genes generated differentially expressed circRNAs

**Fig. 5:**
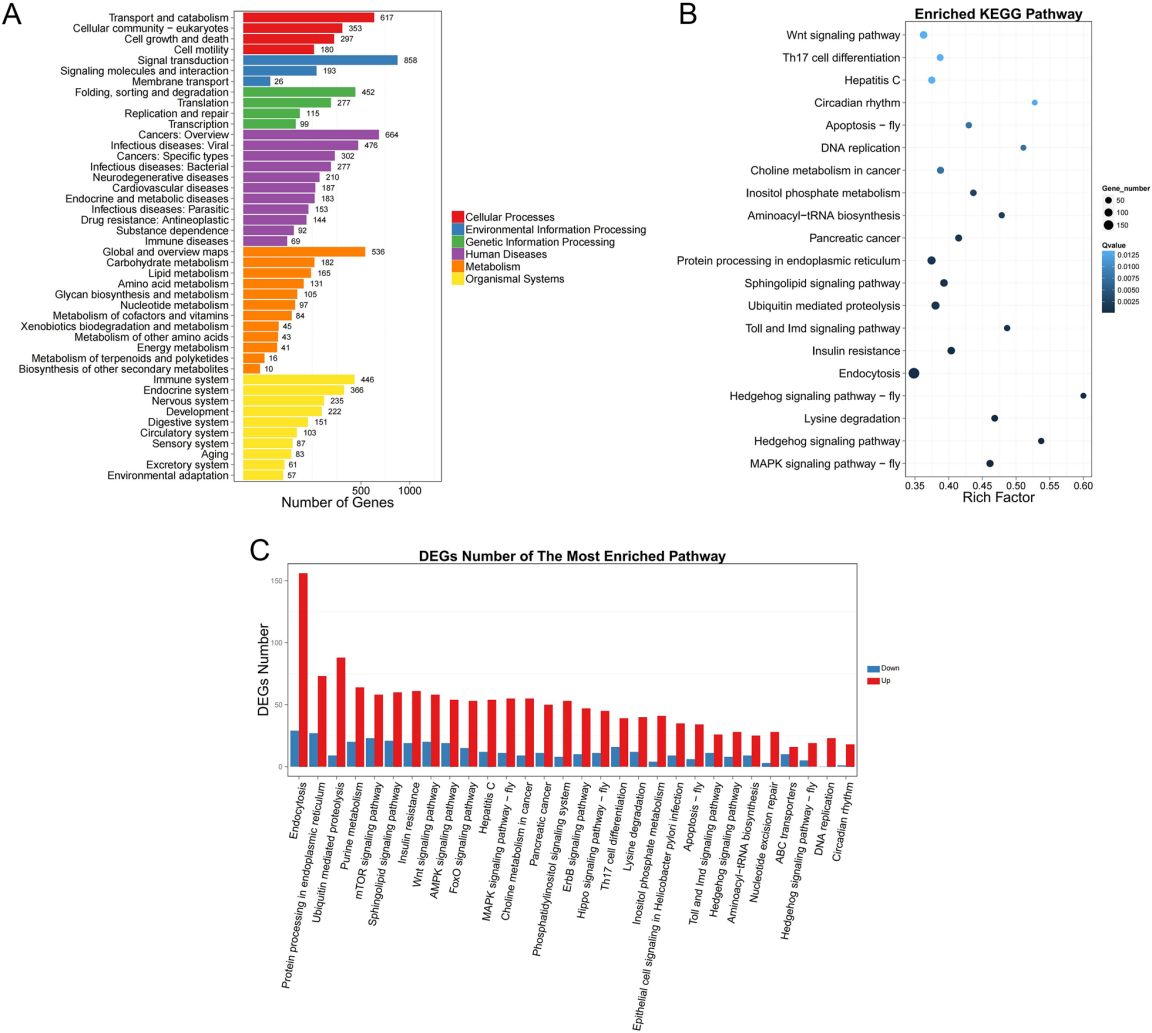
Pathway function analysis of gene generated circRNAs that were differentially expressed. **A** Pathway classification of gene generated circRNAs that were differentially expressed. **B** Pathway enrichment results of gene generated circRNAs that were differentially expressed. **C** Enrichment pathway statistics of genes that differentially expressed circRNAs

Based on the results of the differential expression analysis, KEGG biological pathway classification and enrichment analysis was performed on genes generated intragenic circRNAs. The genes were divided into seven branches according to the KEGG metabolic pathways involved, namely cellular processes, environmental information processing, genetic information processing, human disease, metabolism, organismal systems, and drug development. The results of pathway classification are shown in Fig. 5A, and the results of pathway enrichment analysis are shown in Fig. 5B. The rich factor is the enrichment factor value, which is the quotient between the number of genes that generated differentially expressed circRNAs and the number of all genes generated circRNAs. The larger the set of data, the more obvious was the enrichment. The statistics of enrichment pathways of up- or downregulated circRNA-derived genes are shown in Fig. 5C.

### The expression level of hsa_circ_0116795 was significantly increased in the peripheral blood of patients with AMI

**Fig. 6:**
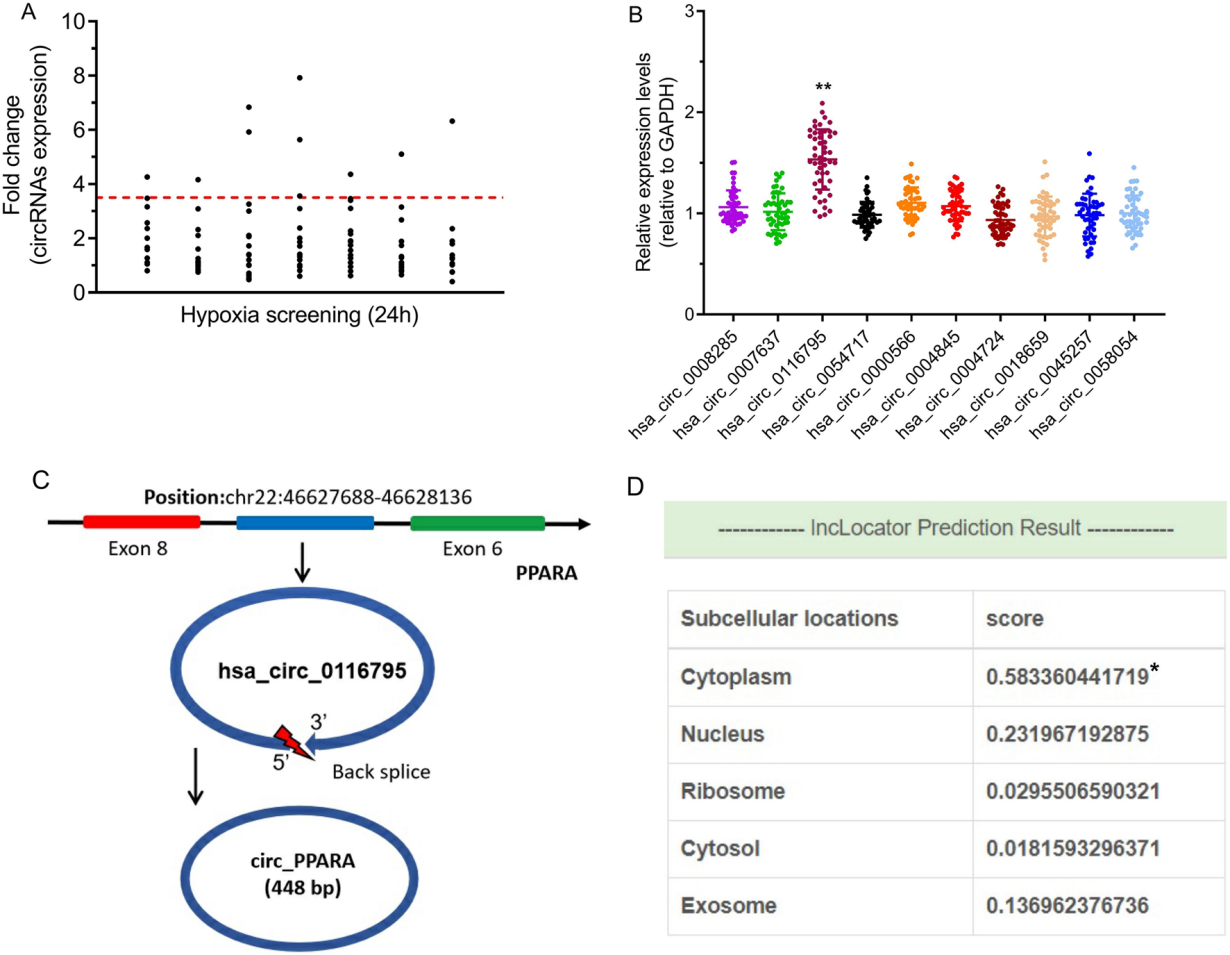
Expression and bioinformatics identification of circ_PPARA in the peripheral blood of patients with AMI. **A** circRNAs that were differentially expressed under 24-h hypoxia conditions in AC16, a human cardiomyocyte cell line. **B** Among the top 10 most highly expressed circRNAs under hypoxic conditions, circ_PPARA was the most overexpressed in the peripheral blood of patients with AMI,* n* = 50. **C** Biological information on circ_PPARA, including its position on chromosomes and transcriptional splicing patterns. **D** Bioinformatics-predicted distribution of circ_PPARA within subcellular structures. *P < 0.05, **P < 0.01

**Fig. 7:**
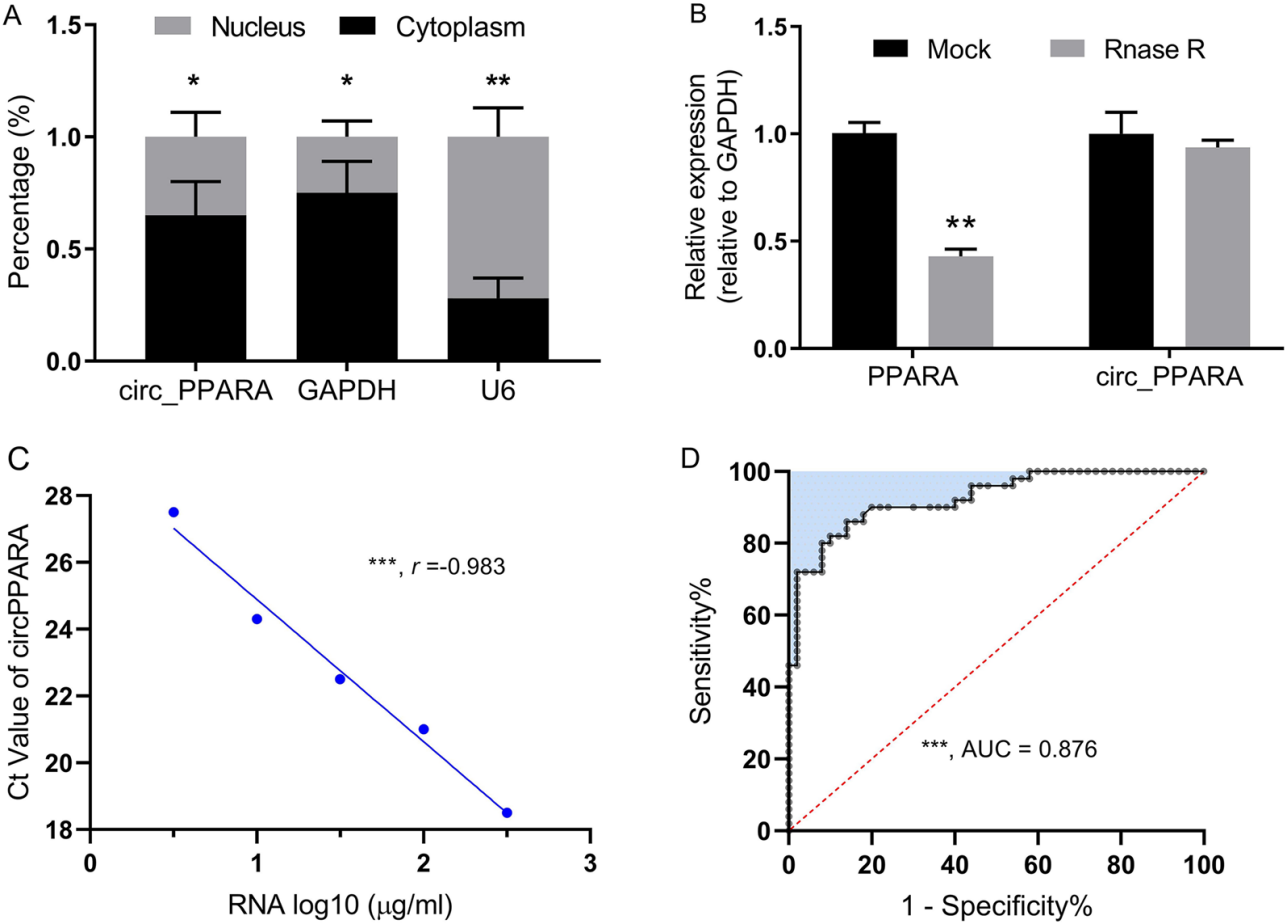
Biological characteristics of AMI-associated circ_PPARA and analysis of its diagnostic utility. **A** Results of nucleocytoplasmic separation experiments confirming that circ_PPARA was mainly located in the cytoplasm. **B** circ_PPARA was resistant to RNA exonuclease. **C** The Ct value of circ_PPARA was inversely proportional to the concentration of total RNA derived from human cardiomyocyte cells in peripheral blood. **D** ROC curve analysis showing that the circ_PPARA level in peripheral blood has potential utility in the diagnosis of AMI. *P < 0.05, **P < 0.01, ***P < 0.001

A human cardiomyocyte hypoxia model was constructed to verify the expression level of the top 100 most highly expressed circRNAs in the above-mentioned high-throughput sequencing data of the patients with AMI. It was found that many circRNAs were significantly highly expressed in cardiomyocytes under hypoxia. By analysing more peripheral blood samples of patients with AMI, we verified the expression of the top 10 most abnormally highly expressed circRNAs in cardiomyocytes under hypoxia and found that hsa_circ_0116795 was the most highly expressed. The schematic diagram in Fig. 6C shows a bioinformatics sequence analysis of hsa_circ_0116795, which is formed by cyclisation (i.e. connection of the 5′ ends and 3′ ends) of the seventh exon of the linear transcript of the parent *PPARA*. Further experiments showed that hsa_circ_0116795 (circ_PPARA) had typical circRNA biological characteristics of resistance to RNase R cleavage and being mainly distributed in the cytoplasm (Figs. 6D and 7A, B).

### Clinical diagnostic value of circ_PPARA levels in the peripheral blood of patients with AMI

We speculated that the overexpression of circ_PPARA in the peripheral blood of patients with AMI was mainly caused by its release from myocardial cells during infarction. To explore this and verify the effectiveness of qRT-PCR-based detection, we added a certain amount of cardiomyocyte-derived RNA into the total RNA of the peripheral blood of healthy individuals, per millilitre and in accordance with a gradient ratio (Fig. 7C), and then used qRT-PCR to detect the Ct value of circ_PPARA. The results showed that as the total RNA from cardiomyocytes increased, the Ct value of circ_PPARA gradually decreased (Fig. 7C), which confirms that the content of circ_PPARA in peripheral blood was effectively detected. The ROC curve analysis based on circ_PPARA levels in the peripheral blood of healthy controls and patients with AMI revealed that the AUC reached 0.876, which could distinguish the patients from the healthy controls to a certain extent (Fig. 7D). These results suggest that circ_PPARA levels in the peripheral blood of patients could be used as a biological marker for the early diagnosis of AMI and for monitoring its progression.

## Discussion

In previous studies on the association between AMI and abnormal expression of circRNAs, there were some shortcomings in the screening strategies used and functional verification performed. In the current study, total RNA was collected from the peripheral blood of five patients with AMI and five healthy controls, circRNAs in these samples were enriched, and the samples were then preliminarily screened by high-throughput sequencing, which revealed thousands of differentially expressed circRNAs (Figs. 1–3). These efforts largely compensated for the shortcomings of the screening methods that have been used in the previous studies mentioned above. That is, previous studies have used microarray chips to screen circRNAs in the blood of patients with AMI. However, as microarray chips are equipped with only a limited number of probes, only dozens of differentially expressed circRNAs have been detected [25]. Our results showed that compared with the peripheral blood of healthy controls, the peripheral blood of the patients with AMI contained significantly more circRNAs with increased expression than circRNAs with decreased expression (Fig. 3A–C). In addition, aside from on the Y chromosome, abnormally highly expressed circRNAs were widely distributed across various chromosomes (Fig. 3D). This suggests that there are widespread abnormalities in gene expression in myocardial cells under hypoxia.

The function of circRNAs is often related to the function of their parent genes [26, 27]. Therefore, we used bioinformatics methods to conduct functional enrichment and pathway enrichment analysis of the genes that were derived from circRNAs that were differentially expressed in the peripheral blood of the patients with AMI (Figs. 4, 5). Gene expression in human tissues is mainly studied at the transcriptional level, and this largely ignores the regulatory factors involved in the translation process. However, Heesch et al. conducted a translatomic analysis of 80 human heart tissues and discovered hundreds of novel microproteins related to circRNA regulation, most of which were located within the mitochondria of myocardial cells [28]. This suggests that circRNAs may play an important role in regulating the hypoxic metabolism of myocardial cells. In the current study, we constructed a model consisting of human cardiomyocytes (AC16 cells) under hypoxia to further explore the expression levels under these conditions of circRNAs that we had identified by high-throughput sequencing (Fig. 6A). As shown in Fig. 6A, dozens of circRNAs were found to be significantly highly expressed in AC16 cells under hypoxic conditions. This suggests that these circRNAs are highly expressed in the peripheral blood of patients with AMI due to the release of myocardial cells into the bloodstream during hypoxic ischaemia or at death. Thus, we collected 50 clinical samples to verify the expression profile of these circRNAs in the patients with AMI. The results showed that hsa_circ_0116795 derived from *PPRA* was significantly highly expressed in these samples (Fig. 6B, C).

AMI is usually caused by coronary atherosclerosis, which leads to the stenosis of the lumen in one or more vessels, resulting in insufficient myocardial blood and oxygen supply coincident with collateral circulation not being fully established [29]. As such, the blood supply is drastically reduced or interrupted, such that severe and persistent ischaemia and hypoxia occurs in the myocardium. If this condition persists for more than 20 min, it can lead to acute myocardial necrosis [29]. Therefore, biomarkers that can predict the pathophysiological state of cardiomyocytes need to be detectable at an early stage, stable, and easily accessible. In this study, we combined bioinformatics and cell biology techniques to identify the biological characteristics of circ_PPARA. The results showed that circ_PPARA is resistant to exonuclease and is mainly present in the cytoplasm of cardiomyocytes (Figs. 6D and 7A, B), which can be easily released when cardiomyocytes are damaged. In molecular cardiology research, circRNAs are emerging as powerful regulators of cardiac disease [30] that may play a key role in myocardial cell proliferation and cardiac repair after myocardial infarction. Huang et al. recently found that downregulation of circular RNA nuclear factor IX can promote myocardial cell proliferation and angiogenesis, inhibit myocardial cell apoptosis, alleviate cardiac dysfunction, and improve prognosis in mice after myocardial infarction [23]. Exploring the molecular mechanisms of circ_PPARA in the pathogenesis of AMI is highly likely to provide new targets and therapeutic strategies for myocardial protection. CircRNAs often participate in disease progression together with miRNAs, but due to their mutual inhibitory effects, the expression levels of miRNAs interacting with highly expressed circRNAs are often low and difficult to detect in peripheral blood. Therefore, we did not include examine such miRNAs in the current study.

We speculated that the peripheral blood of patients with AMI contains circ_PPARA as it is released during the rupture of myocardial cells after ischaemia and hypoxia or is secreted by such cells. To explore this speculation and verify the validity of the detection method we used, we added different doses of human cardiomyocyte total RNA to the total peripheral blood RNA of healthy controls to form mixed RNA samples and found that the copy number of circ_PPARA in these samples was significantly correlated with the dose of human cardiomyocyte total RNA (Fig. 7C). These results suggest that the level of circ_PPARA in the peripheral blood of patients with AMI somewhat reflects the extent of damage to their myocardia. ROC curve analysis showed that the circ_PPARA level in the peripheral blood of the patients with AMI was a good diagnostic reference and may be useful as a clinical biomarker due to its good detection rate and a low false-positive rate for disease identification (Fig. 7D). However, the sample size in this study was rather small, and no external, independent validation was performed. Therefore, future studies should use a larger sample size and conduct multicentre clinical cohort validation to develop new circRNA biomarkers for AMI.

## Conclusion

In summary, we demonstrated that high-throughput sequencing is an effective method for screening disease-related circRNA markers in the peripheral blood of patients with AMI. Our results showed that many circRNAs are abnormally expressed in the peripheral blood of patients with AMI and that hypoxia of AC16 cells can induce the high expression of circ_PPARA that is highly resistant to exonuclease. Overall, this study reveals that the expression level of circ_PPARA in the peripheral blood of patients with AMI has significant potential utility for early diagnosis of AMI, and is likely to be a new biomarker of this disease.

### Supplementary Information

**Additional file 1: Table S1.** Sequence of primers.

**Additional file 2: Table S2.** Biological information of all circRNAs differentially expressed between the healthy controls and patients with AMI.

## Data Availability

The data that support the findings of this study are available from the corresponding author upon reasonable request.
