# Supplementary material for: Hypoxia-induced circRNAs encoded by PPARA are highly expressed in human cardiomyocytes and are potential clinical biomarkers of acute myocardial infarction
Source: Eur J Med Res. 2024 Mar 12;29:159. doi: 10.1186/s40001-024-01753-3 (PMC10929223; doi:10.1186/s40001-024-01753-3)
Supplement: Supplementary file 1 — Additional file 1: Table S1. Sequence of primers. [file 40001_2024_1753_MOESM1_ESM.doc]

Table S1 The sequence of primers

| **Name** | **Sequence** |
| --- | --- |
| hsa_circ_0008285 primer-F | 5'-GGCCCAGGATACACCCACTA-3' |
| hsa_circ_0008285 primer-R | 5'-AGCCTTTCCACCGAACCAAA-3' |
| hsa_circ_0007637 primer-F | 5'-GTTTCCCCGCAAATGACTGG-3' |
| hsa_circ_0007637 primer-R | 5'-TCTGGAACAAGGTTCCCACTG-3' |
| hsa_circ_0000566 primer-F | 5'-TGGACCTCAGTGTTGTGGAG-3' |
| hsa_circ_0000566 primer-R | 5'-CTCCAACTGCAAATTGTTCTGC-3' |
| hsa_circ_0054717 primer-F | 5'-AGCAGCTGTATAGCATCTTCACT-3' |
| hsa_circ_0054717 primer-R | 5'-GTGAAGAGCCACAGTGGGAA-3' |
| GAPDH primer-F | 5'-AGAAGGCTGGGGCTCATTTG-3' |
| GAPDH primer-R | 5'-AGGGGCCATCCACAGTCTTC-3' |
| PPARA primer-F | 5'-TTCGCAATCCATCGGCGAG-3' |
| PPARA primer-R | 5'-CCACAGGATAAGTCACCGAGG-3' |
| U6 primer-F | 5'-CTCGCTTCGGCAGCACA-3' |
| U6 primer-R | 5'-AACGCTTCACGAATTTGCGT-3' |
| hsa_circ_0116795 primer-F | 5'-GTTCAATGCACTGGAACTGGA-3' |
| hsa_circ_0116795 primer-R | 5'-CCAGCGTCTTCTCAGCCATA-3' |
| hsa_circ_0004845 primer-F | 5'-GCTGCCGGGACATGTATGAG-3' |
| hsa_circ_0004845 primer-R | 5'-TGTGCCACAAAGGCTTGGTTT3' |
| hsa_circ_0004724 primer-F | 5'-TCAAAAATCCCGGGACCAGC-3' |
| hsa_circ_0004724 primer-R | 5'-ACTGGTGAACCATCTCTACACG-3' |
| hsa_circ_0018659 primer-F | 5'-CACACCTCGAGACAAGCAGA-3' |
| hsa_circ_0018659 primer-R | 5'-GTAGGCCCTCAAATGGGGTG-3' |
| hsa_circ_0045257 primer-F | 5'-TTGCCCTTTGGAGCTTGGTG-3' |
| hsa_circ_0045257 primer-R | 5'-CCTTGCTGGGACTGAACGTA-3' |
| hsa_circ_0058054 primer-F | 5'- AGCTGTTGCCCAATATGGCT-3' |
| hsa_circ_0058054 primer-R | 5'- ACTTCGAGGGCTAAACCACA-3' |

# 
